# Morphological Response in Cancer Spheroids for Screening Photodynamic Therapy Parameters

**DOI:** 10.3389/fmolb.2021.784962

**Published:** 2021-11-18

**Authors:** Jose R. Aguilar Cosme, Dan C. Gagui, Helen E. Bryant, Frederik Claeyssens

**Affiliations:** ^1^ Department of Materials Science and Engineering, Kroto Research Institute, University of Sheffield, Sheffield, United Kingdom; ^2^ INSIGNEO Institute for in Silico Medicine, University of Sheffield, Sheffield, United Kingdom; ^3^ Department of Oncology and Metabolism, The Medical School, University of Sheffield, Sheffield, United Kingdom

**Keywords:** photodynamic therapy, cancer spheroids, screening, morphometric, image analysis

## Abstract

Photodynamic therapy (PDT) is a treatment which uses light-activated compounds to produce reactive oxygen species, leading to membrane damage and cell death. Multicellular cancer spheroids are a preferable alternative for PDT evaluation in comparison to monolayer cell cultures due to their ability to better mimic *in vivo* avascular tumour characteristics such as hypoxia and cell-cell interactions, low cost, and ease of production. However, inconsistent growth kinetics and drug responsiveness causes poor experimental reproducibility and limits their usefulness. Herein, we used image analysis to establish a link between human melanoma C8161 spheroid morphology and drug responsiveness. Spheroids were pre-selected based on sphericity, area, and diameter, reducing variation in experimental groups before treatment. Spheroid morphology after PDT was analyzed using AnaSP and ReViSP, MATLAB-based open-source software, obtaining nine different parameters. Spheroids displayed a linear response between biological assays and morphology, with area (R^2^ = 0.7219) and volume (R^2^ = 0.6138) showing the best fit. Sphericity, convexity, and solidity were confirmed as poor standalone indicators of spheroid viability. Our results indicate spheroid morphometric parameters can be used to accurately screen inefficient treatment combinations of novel compounds.

## Introduction

Photodynamic therapy (PDT) is an FDA-approved cancer treatment which produces reactive oxygen species through the specific excitation of a photosensitizer (PS) at a given wavelength. It is multifactorial, requiring the evaluation of parameters like irradiation wavelength, light dose, and drug concentration (Cho et al., 2013). High-throughput screening has been explored as a strategy for simultaneously evaluating multiple PDT parameters, which has shown great success in cell monolayers (Lou et al., 2010). However, PDT evaluation in monolayers is a poor indicator of treatment effectiveness due to their homogeneous conditions and large surface area (Brüningk et al., 2020). In contrast, 3D cell culture models replicate *in vivo* conditions such as hypoxia, dormancy, and cell-cell interactions more accurately than 2D models. Multicellular cancer spheroids (MCTS) can replicate *in vivo* conditions such as hypoxia, dormancy, and cell-cell interactions more accurately, making them an ideal model for evaluating PDT parameters (Imamura et al., 2015).

Spheroids can be grown using a variety of methods, ranging from hanging drop and rotating vessels, to scaffolds with non-adherent surfaces. A significant challenge in spheroid-based platforms for drug screening is the inherent variability found between spheroids, regardless of consistency in growth conditions. Recent advances in culture techniques include spinner flasks, rotary culture vessels, and microfluidic devices, which aim to tightly control spheroid growth. However, maintenance of these systems is both expensive and time-consuming (Mehta et al., 2012). The agar coating method with 2-hydroxyethylagarose has been previously used to successfully produce MCTS from various cell lines, such as NCI-ADR-RES (ovarian adenocarcinoma) and HUH7 (hepatocellular carcinoma) (Perche and Torchilin, 2012; Rühland et al., 2015). This is a simple, low-cost, and reliable method for cultivating spheroids which makes use of agarose dissolved in serum-free media, though it frequently leads to heterogeneous spheroid morphology.

Minor variations in spheroids are caused by edge effects from uneven agarose surfaces within individual wells and evaporation-induced liquid media loss at the plate periphery (Das et al., 2016). Effective control and monitoring of spheroid morphology remain a challenge with most production methods, particularly for high-throughput screening applications in which hundreds of samples are used simultaneously (Sant and Johnston, 2017). It should be noted that spheroid growth and behavior is highly specific to each cell line, with some requiring supplements such as reconstituted basement membrane (rBM) to form cohesive spheroids and impacting *in vitro* models as protein expression changes (Ivascu and Kubbies, 2007). These variations are also present depending on the method of spheroid production, use of co-cultures, and integration of other devices such as microfluidics for analysis.

Drug screening using spheroids has seen many different approaches including the evaluation of morphology through optical coherence tomography (OCT), confocal microscopy, light sheet fluorescence microscopy to monitor drug penetration, image cytometry for apoptosis quantification, and electrical impedance monitoring of membrane integrity in polydimethylsiloxane (PDMS) microdevices (Kessel et al., 2017; Curto et al., 2018; Hari et al., 2019; Lazzari et al., 2019). Spheroid morphology has been found to be a key parameter in experimental standardization, ensuring other factors like microenvironment to be more similar between samples. These parameters (i.e., diameter, volume, sphericity, etc.) can be obtained through various types of microscopy, such as light, fluorescence, and light sheet microscopies (Sant and Johnston, 2017). They can then be used to observe variations in growth kinetics and improve experimental reproducibility (Friedrich et al., 2009).

Spheroids are known for being highly susceptible to variations between samples and within repeats, being affected by parameters such as partial oxygen pressure, compactness, diffusion, and nutrient gradients (Kim et al., 2010). In some cases, conventional methods for evaluating 2D cell cultures have been shown to be unsuitable for 3D cell cultures, further increasing variability and leading to uncertainty about the validity and reproducibility of acquired data (Kepp et al., 2011). Thus, there is a lack of standardized, easily accessible methods tailored for MCTS which can to provide quantification of individual sample quality and drug-responsiveness (Vinci et al., 2012). Ideally, such protocols would be adaptable for high-throughput screening (HTS) applications, where thousands of chemical compounds are tested with standardized conditions.

Recently, computer-assisted models have gained increased interest as complementary tools to *in vitro* assays for predicting spheroid growth kinetics and determination of the biological effect of various compounds (Costa et al., 2016). This is particularly advantageous for HTS as large image sets can be rapidly evaluated. Nonetheless, this software is centered on high-throughput screening, which relies on costly automated equipment and limits usage by non-specialised users. The development of open-source alternatives is a key step in the introduction of reliable computer-assisted image analysis to researchers working with MCTS. Piccinini et al. (2015) developed AnaSP (ANAlyse SPheroids) and ReViSP (Reconstruction and Visualization from a Single Projection), MATLAB-based tools for analyzing various spheroid parameters using widefield microscopy images (Piccinini, 2015; Piccinini et al., 2015).

In this study, we propose a method to improve drug screening using image-based analysis of spheroid morphometric parameters. We demonstrate a reduction of variation in groups by pre-selecting spheroids with high sphericity, area, and diameter before use with *in vitro* experiments. Spheroids subjected to photodynamic therapy were used to monitor changes in morphology before and after treatment, showing extensive damage after 24 h. Image processing was significantly affected by cell debris after treatment, requiring removal to improve automatic spheroid segmentation. Morphometric parameters area, volume, and diameter were shown to have a linear correlation with decreased viability and dsDNA content. Conversely, sphericity and convexity were determined to be independent of spheroid damage. Our results show the potential of image-based analysis for 3D cell culture models to complement *in vitro* data and highlight key areas of opportunity for their use in drug screening.

## Materials and Methods

### Materials

Citric acid monohydrate, sucrose, ethylenediamine, protoporphyrin IX, sodium chloride, (N-(3-Dimethylaminopropyl)-N′-ethylcarbodiimide), N-Hydroxysuccinimide, 2-hydroxyethylagarose, formaldehyde, perinaphthenone, 2-(N-Morpholino) ethanesulfonic acid, acetone, dimethyl sulfoxide, 2-mercaptoethanol and N,N-dimethylformamide were acquired from Sigma Aldrich (United Kingdom). Dulbecco’s modified Eagle’s medium (DMEM, high glucose), Dulbecco’s modified Eagle’s medium (DMEM, high glucose, without phenol red), foetal bovine serum (FBS), Quant-iT Picogreen dsDNA quantification kit, Pierce LDH cytotoxicity assay kit, and trypsin-EDTA were obtained from Thermo Fisher (United Kingdom). Syringe filters with a 0.2 μm pore size were acquired from Sarstedt (United Kingdom). 1 KDa MWCO, 6.4 ml/cm dialysis tubing was acquired from Spectrum Labs (United States of America). All chemicals were used as received unless stated otherwise. Deionized water was used for all buffers and samples in experiments. Septa steel ring caps and 35 ml glass reaction vessels were obtained from CEM Corporation (United Kingdom).

### Spheroid Culture

Human melanoma cells (C8161) were cultured in phenol red-free DMEM with 10% foetal calf serum, 1% penicillin and streptomycin, and 1% L-glutamine. Cells were cultured in a T75 plate at 37°C, 5% CO_2_ until around 80% confluence. Multicellular tumour spheroids were produced utilizing the agar overlay method. A 1.5% agarose solution was prepared with 2-hydroxyethylagarose and standard cell culture media (DMEM). This solution was sterilized by autoclave and stored at 4°C until used. Agar-coated plates were prepared by adding 100 μl of the agarose solution into each well and left to set at room temperature for at least 1 h. Plates were seeded with 100 μl phenol red-free media containing 1.2 × 10^4^ cells per well and returned to the incubator until spheroids reached approximately 500 μm diameter. Growth media was changed every third day by adding 100 μl to each well, followed by the removal of an equal volume.

### Evaluation of Spheroid PDT Response

Protoporphyrin IX (PpIX) and a carbon dot conjugate (PpIX-CD) were used to produce phototoxicity. PpIX-CD was synthesized according to our previously established protocol (Aguilar Cosme et al., 2019). Samples were processed with a Hieschler UP50H ultrasonic probe prior to the dilution to remove aggregates. These solutions were dissolved in phenol red-free media (50 μg/ml) and were kept refrigerated until used. Stock solutions were placed in an ultrasonic water bath for 15 min at 37°C before adding to spheroids.

**FIGURE 1:**
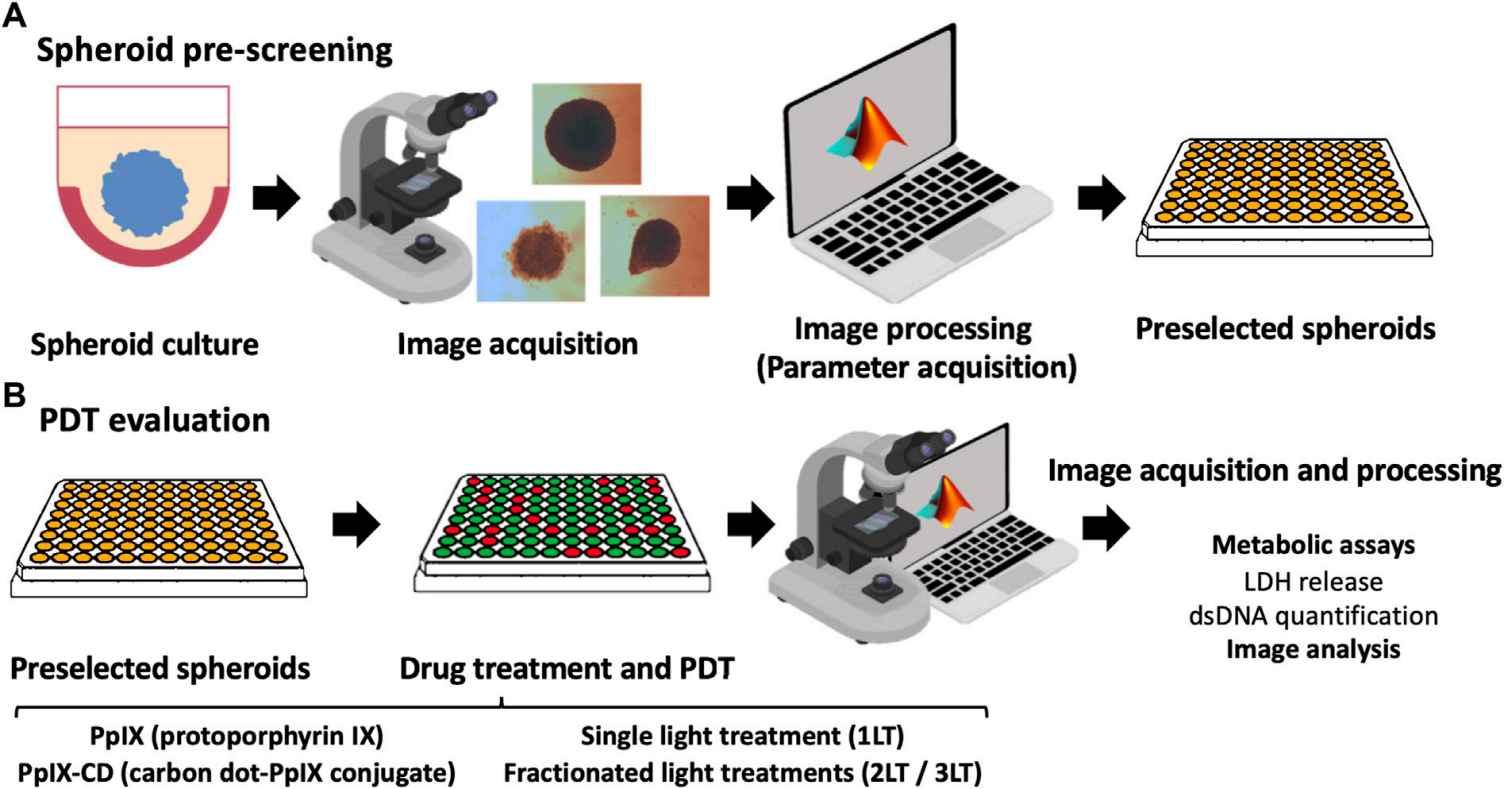
Summary of PDT parameter screening workflow. Spheroids are cultured using a 2-hydroxyethylagarose surface coating to prevent adhesion and left to grow until reaching around 500 µm in diameter. Individual spheroids were monitored and pre-selected based on their morphology using the parameters of sphericity, area, and equivalent diameter **(A)**. Selected spheroids were then incubated with PS and subjected to PDT. Widefield microscopy images were obtained after drug treatment using a 4X objective, clearing cell debris from each well to improve data acquisition. Morphometric parameters were extracted from the images and compared to results from biological assays (LDH release and total DNA content) **(B)**. Further information could be obtained for parameter screening, such as protein expression or advanced microscopy.

Photosensitiser concentrations of 5 and 10 μg/ml were used to treat samples by adding stock solutions to achieve desired concentrations in each well. Plates were incubated for 2 h within the incubator and PS-supplemented media was replaced by fresh media before PDT. Spheroids were subjected to single, double, and triple light exposure periods using a M405L2 ThorLabs mounted LED with a collimator adapter (405 nm, 2.76 mW/cm^2^) to induce ultra-low fluence PDT. Fluence was set at 5 J/cm^2^, corresponding to 30 min under light exposure based on our previous work with spheroids (Aguilar Cosme et al., 2021). The effect of sequential fractionated light exposures was evaluated by quantifying LDH release and DNA quantification in treated spheroids 24 h after the initial light dose, ranging from single (1LT), double (2LT), and triple (3LT) light treatments (Figure 1).

#### LDH Release Assay

LDH release was measured in all samples by collecting 50 μl of media and transferring it to a 96-well plate. Spheroids with no conjugates and equal irradiation times were used as negative controls for spontaneous LDH release. The positive control was carried out by incubating spheroids with TE buffer (10 mM Tris-HCl, 1 mM EDTA, pH 7.5) for 45 min. An additional four freeze-thaw cycles were used to ensure membrane disruption and indicate maximum LDH release. Subsequently, 50 μl LDH working solution was added to each well and covered to avoid contact with light. Plates were incubated for 30 min and 50 μl LDH stop solution was added to finalize the reaction. Absorbance for each well was read at 490 nm (LDH) and 680 nm (background) with a with a fluorescence plate reader (Biotek Instruments ELx800). Viability was calculated with the following formula:

$$\text{\%}\mathrm{Cytotoxicity}=\;\frac{\left(\mathrm{Sample}\;\mathrm{LDH}\;\mathrm{release}-\mathrm{Spontaneous}\;\mathrm{LDH}\;\mathrm{release}\right)}{\left(\mathrm{Maximum}\;\mathrm{LDH}\;\mathrm{release}-\mathrm{Spontaneous}\;\mathrm{LDH}\;\mathrm{release}\right)}\times 100$$

$$\text{\%}\mathrm{Viability}=\text{\%}\mathrm{Cytotoxicit}y_{\mathrm{Control}}\;-\text{ \%}\mathrm{Cytotoxicit}y_{\mathrm{Sample}}$$

#### dsDNA Quantification Assay

PicoGreen (PG) working solution was prepared by dissolving the reagent in TE buffer (10 mM Tris-HCl, 1 mM EDTA, pH 7.5) according to the instructions from the manufacturer. Spheroids were removed from each well and placed in a 96-well plate and carefully washed three times with 50 μl sterile PBS to remove cellular debris. Cell lysis was performed by adding 50 μl TE buffer to each well and freeze-thawed four times. An equivalent volume of 100 μl PG working solution was added. Plates were covered from light and incubated for 10 min at room temperature. Fluorescence was read at 485 nm excitation and 528 nm emission with a fluorescence plate reader (Biotek Instruments FLx800). A blank was prepared by adding deionized water and PG in equal volumes. %dsDNA was calculated the following formula:

$$\text{\%}\mathrm{dsDNA}=\;\frac{\left(\mathrm{Sample}\;\mathrm{dsDNA}-\mathrm{Control}\;\mathrm{dsDNA}\right)}{\mathrm{Control}\;\mathrm{dsDNA}}\times 100$$

### Automated Image Analysis With AnaSP

MCTS were imaged using an AE2000 inverted light microscope (Motic, United States) fitted with a Moticam 2.0 camera (2.0 MP) and a 4× objective. Images were taken before and after clearing cellular debris from each well. White balance was used increase spheroid contrast against the background. AnaSP version 1.2 (https://sourceforge.net/projects/anasp/) was run with MATLAB R2019b (Version 9.7) with Image Processing Toolbox. The standard parameters were extracted: Area, Convexity, Equivalent Diameter, Length of Major Diameter Through Centroid, Length of Minor Diameter Through Centroid, Perimeter, Solidity, Sphericity, and Volume.

### Statistical Analysis

Experiments carried out with three independent repeats in sextuplicate (*N* = 3, *n* = 6) and results were normalized using untreated spheroids placed outside incubation conditions as controls. Statistical analysis was carried out using GraphPad Prism version 8.3.0. The comparison of metabolic activity was evaluated by 2-way ANOVA analysis with Dunnett’s test for multiple comparisons, with adjusted *p* values < 0.05 considered statistically significant. Data was presented as means ± SD (standard deviation). Linear regression lines were set to intercept at y = 0, indicating total spheroid disruption (shown as 0% viability or dsDNA content of the control).

## Results

### Monitoring of Individual Spheroid Variability

The open-source software AnaSP/ReViSP can extract morphological parameters from spheroids by processing suitable images and identifying the area of interest based on histogram intensity and automatic triangle segmentation. Calculated morphological parameters are listed in Supplementary Table S1. Image pre-processing is an essential step in parameter acquisition as it influences all obtained data. Drug-treated spheroids were rinsed with PBS to remove cell debris accumulated at the bottom of each well to improve segmentation. While manual segmentation can be done through free-hand drawing using a stylus or mouse cursor, results are significantly different compared with automatic segmentation and are highly variable between users. Manual segmentation significantly reduced all parameters except for those based on spheroid roundness. Sphericity and convexity were overestimated using manual segmentation by 13.92 and 17.69%, respectively. In comparison, all other parameters decreased, with area (+28.25%) and volume (+39.87%) being the most changed (Supplementary Table S2). Therefore, sample images were only utilized in data analysis if automatic image segmentation was successful.

The non-adherent surface overlay method facilitated cell aggregation after seeding, rapidly forming spheroids which reached approximately 500 µm in diameter before use in experiments. Spheroids started as slightly irregular spherical masses that progressively became more round as cells compacted and proliferated on the outer layers. Growth continued until stagnating at around 600 µm diameter, with most samples maintaining their sphericity throughout their growth. Occasionally, some spheroids develop unusual morphologies during growth. These samples were excluded from further analysis (Supplementary Figure S1).

**FIGURE 2:**
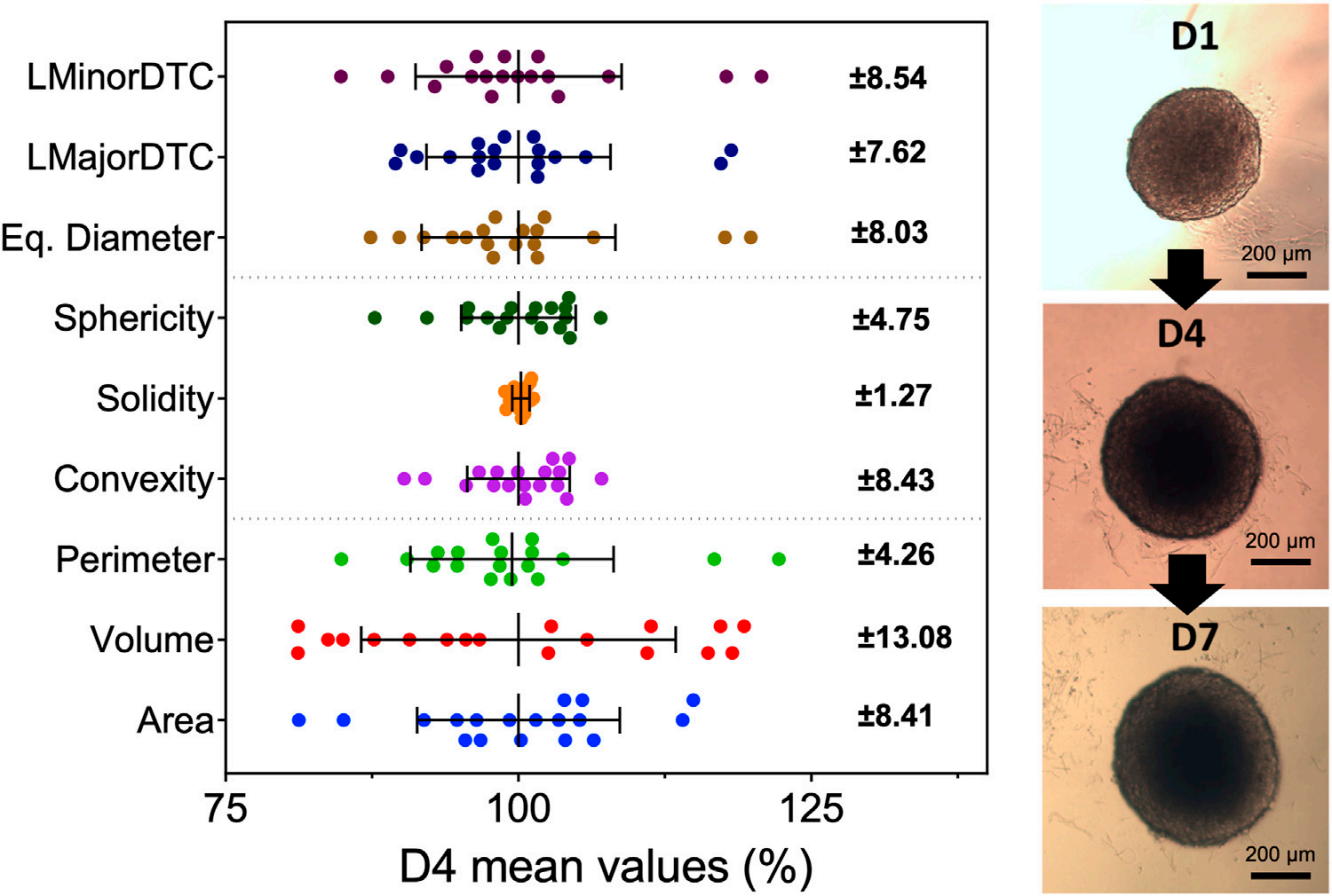
Spheroids show variability in all morphometric parameters at all time points during growth. Parameters were obtained with widefield microscopy images and AnaSP with spheroids at day 4 after seeding (*N* = 3, *n* = 6). Error bars represent standard deviation from the mean value of all samples (shown on the right). Each point represents a single spheroid before selection for PDT. Sphericity, area, and equivalent diameter were chosen to pre-select samples before continuing to *in vitro* PDT evaluation. Although solidity had low variation, it was not possible to distinguish between healthy and damaged samples. Spheroids continued to grow and maintain their morphology past day 7, reaching around 600 µm in diameter.

Spheroids grown in identical conditions showed slight variability for all morphometric parameters acquired with AnaSP, which was expected due to irregularities in agar covering. Volume showed the highest variation likely due to its calculation using an estimated projection from a 2D image with ReViSP. Morphological parameters were mostly clustered near the group mean. Solidity was the least variable measurement in the control, though this parameter was not used for further comparisons as PDT-damaged spheroids showed high variation (0.5–0.99 compared to 0.81–0.99 in controls). Results show various morphometric parameters can be used to reduce variation in sample groups through pre-selection before *in vitro* experiments, shown in Figure 2. Spheroid selection should be mostly based on samples with sphericity higher than 0.8 as it has been shown to impact diffusion kinetics. In addition, the removal of spheroids with outlying values for parameters such as area and equivalent diameter can further improve pre-selection, resulting in groups of highly similar spheroids (Supplementary Figure S2).

### Monitoring of Post-PDT Spheroid Morphology

**FIGURE 3:**
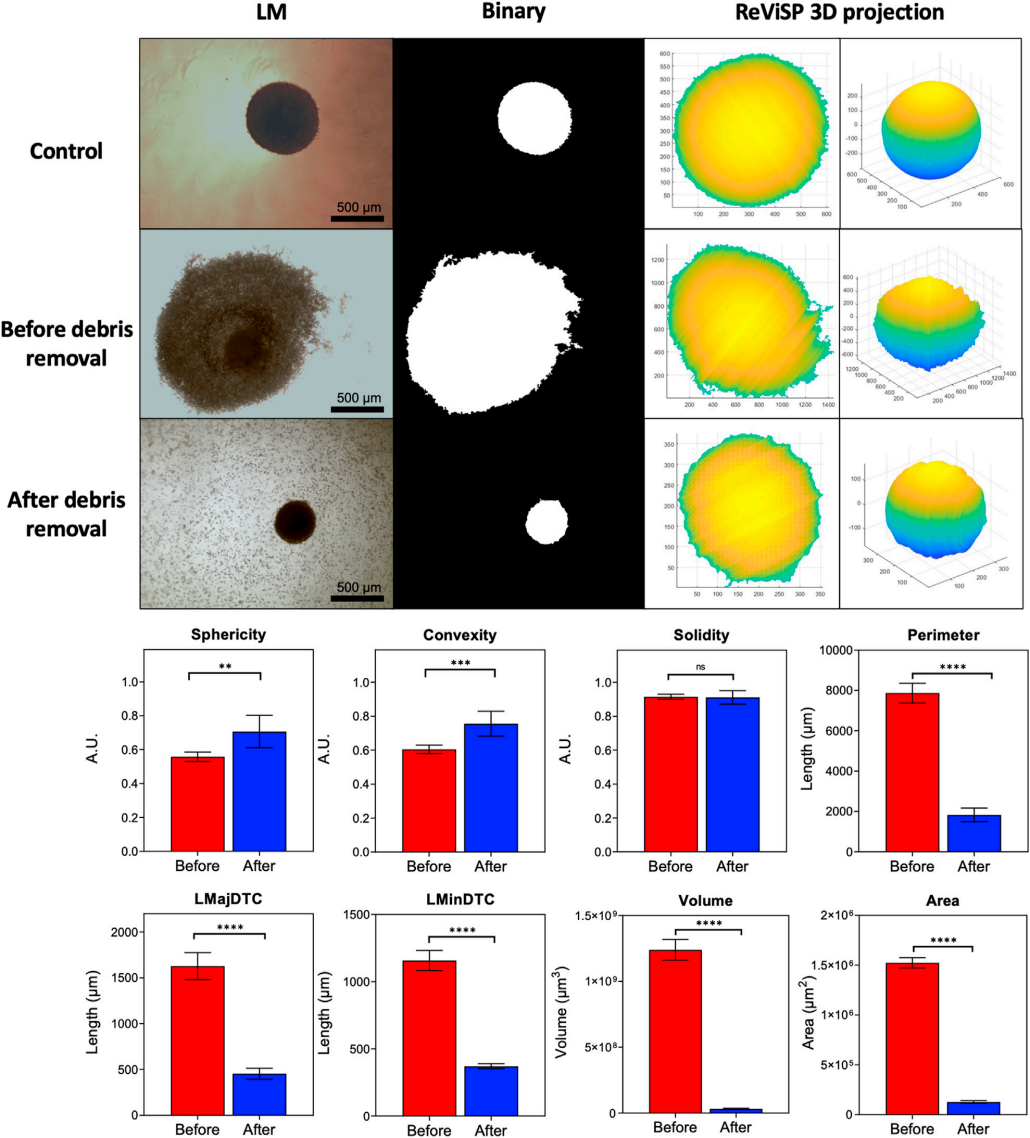
Parameter extraction improves as debris is cleared from the well. Automatic segmentation depends on initial widefield light microscope image quality. Conversion to binary causes accuracy to decrease as cellular debris accumulating in the well bottom is mistaken for spheroid mass. Debris removal significantly changes extracted parameters. Area and volume are highly affected as they depend on 3D projections from the binary mask obtained with AnaSP and ReViSP. (*N* = 3, *n* = 6).

PDT caused significant damage to spheroids, seen as sloughing of outer layers and accumulation of cell debris. Parameter acquisition was significantly affected by the obscured foreground and background within images, with cell debris being identified as part of the main spheroid mass after automatic segmentation (Figure 3). We compared images from spheroids after PDT (2.5 μg/ml PpIX, 5 J/cm^2^, 1LT) before and after debris removal to determine key differences in measurements. Sphericity and convexity increased after well rinsing, though solidity remained constant. In comparison, all other parameters were significantly reduced, with area and volume showing the greatest change.

### Selecting Parameters for PDT Evaluation

**FIGURE 4:**
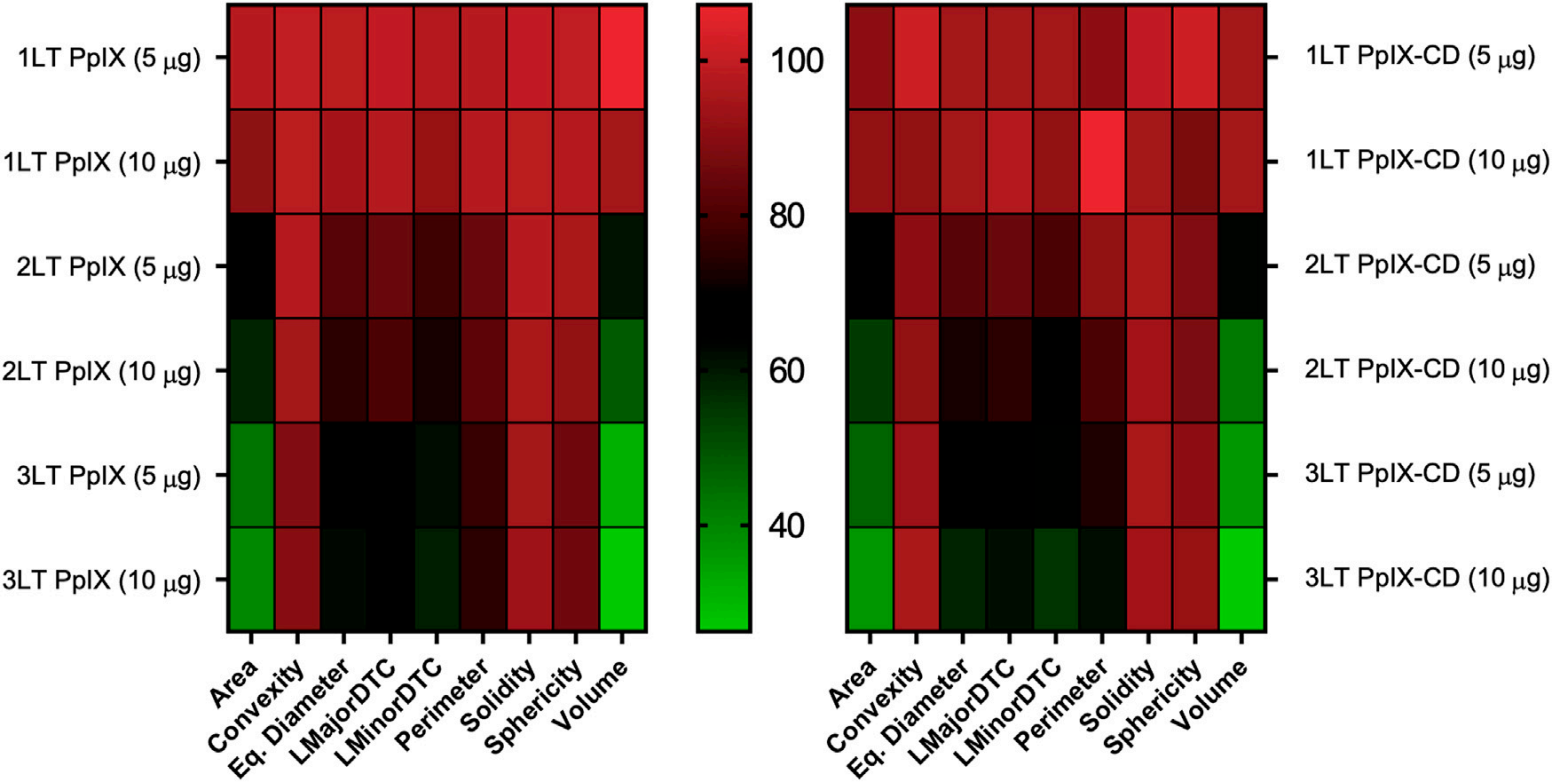
Variability in spheroid morphology at 24 h post-PDT. Parameters are compared to untreated spheroids, shown as 100% of each value. Each square corresponds to nine independent repeats. Area and volume show the greatest variations between PDT treatment conditions in both PpIX and PpIX-CD, followed by diameter-related parameters. Spheroid roundness (convexity, solidity, sphericity) did not show high variation after photoinduced damage. (*N* = 3, *n* = 6).

Spheroids were treated using a combination of drug doses and light treatments with varying degrees of damage reflected in their morphometric parameters. Greater damage was achieved by increasing both drug concentration and light exposures (1LT-2LT) resulting in significantly reduced size after treatment. We used these results to select the best parameters for observing spheroid response to PDT compared to untreated samples (Figure 4). The condition that was most effective was 10 µg PS (3LT), which showed a reduction of up 65% of the spheroid mass at 24 h post exposure. In comparison, lower combinations such as 5 µg (1LT) did not show significant change from untreated spheroids. Parameters based on sphericity did not show significant change regardless of treatment conditions. In contrast, diameter and area-based measurements showed greater change, particularly with 3LT. Area and volume were shown to be highly useful parameters to differentiate treatments as they showed variation across all combinations. Values for area and volume ranged from 16.21—103.79% of the control values compared to the range of 45.17–113.19% of LMajorDTC, LMinorDTC, and Eq. Diameter.

### Comparing Morphometric Parameters to Biological Assays

**FIGURE 5:**
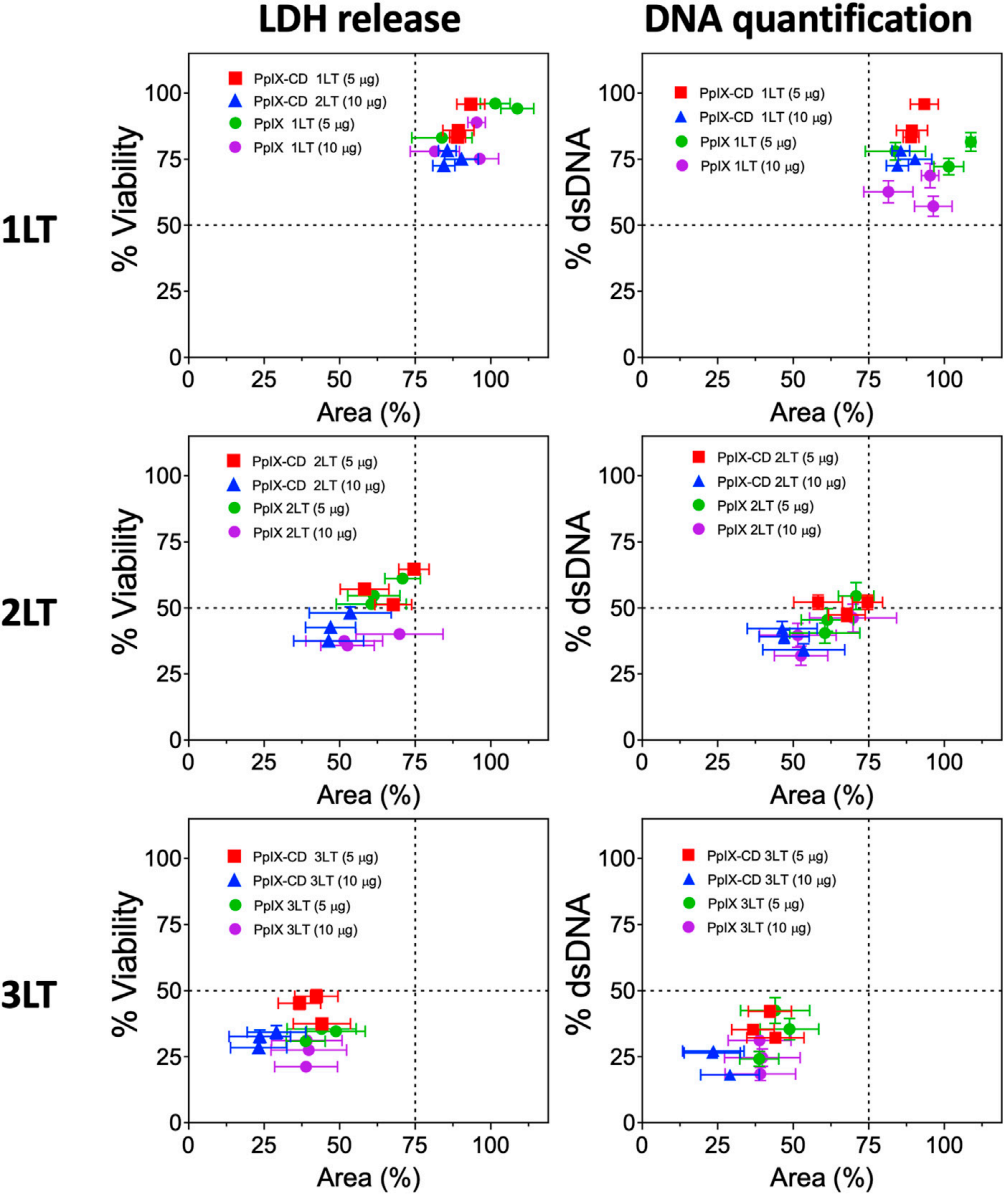
PDT treatment combinations lead to varying degrees of spheroid damage. PpIX and PpIX-CD show similar reductions to viability and spheroid area as light treatments (LT) were increased. Light fractionated treatments (2LT and 3LT) caused greater damage to spheroids, reaching around 25% viability and area in comparison to single light exposure (1LT). Each point represents a spheroid group with different PDT conditions (*N* = 3, *n* = 3). Similar combinations (e.g., PpIX 3LT 5 µg and 10 µg) could not be differentiated.

PpIX and PpIX-CD showed effective inhibition of spheroid growth after PDT, indicated by decreased viability and dsDNA content. Spheroids rapidly showed cell death which could be seen as accumulating cell debris, though sphericity remained largely unchanged regardless of treatment conditions. Assay data plotted against spheroid area showed treatments could be separated according to number of light treatments. Drug dose did not significantly affect spheroid morphology, though viability was reduced. Figure 5 shows 1LT conditions can be separated from 2LT and 3LT-based treatments using only area as the primary indicator of PDT-induced damage. Spheroids showed variation between experimental groups using identical conditions, with variations in both morphology and viability (Supplementary Figure S3). These differences occurred regardless of pre-selection based on morphology. distinguishing between two very similar treatments, such as 2LT (10 μg) and 3LT (5 μg) proved to be impractical. Combinations that showed a reduction in area under 25% (75% of control spheroid area) appeared to be significantly less effective at reducing both viability and DNA content.

**FIGURE 6:**
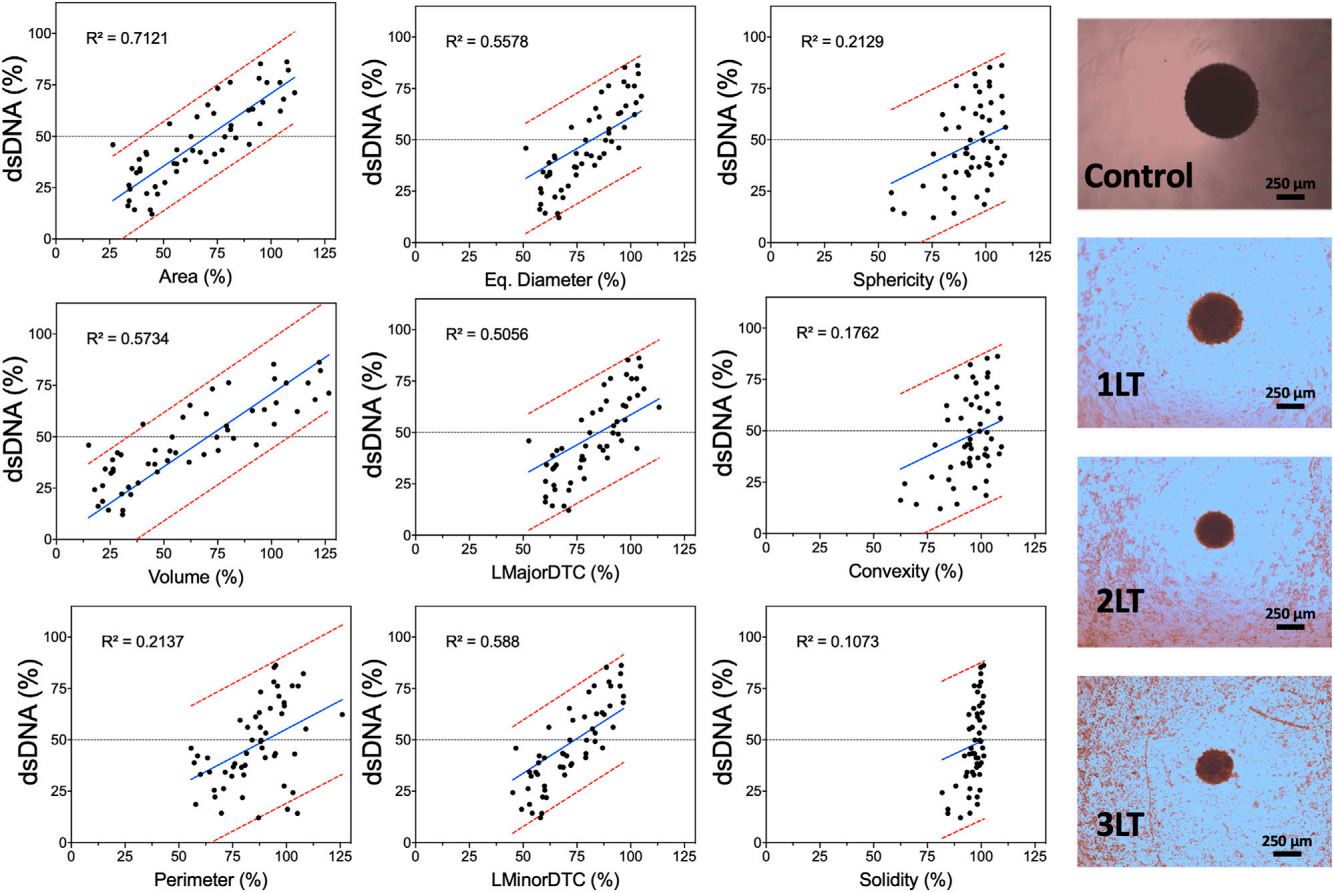
Morphometric parameters can be used to predict PDT-induced damage in spheroids. Acquired parameters show varying degrees of linear correlation with LDH release based on control spheroids at the same time point (24 h after light activation). Each dot represents a single spheroid after PDT with PpIX or PpIX-CD. The regression line is shown in blue, and the 95% prediction intervals are shown in red. Widefield images of spheroids after different combinations of PDT treatments are shown on the right (PpIX, 10 µg/ml). (*N* = 3, *n* = 6).

The goodness of fit parameter (R^2^) showed a linear correlation between single spheroid response to PDT and various morphometric parameters. Various morphometric parameters have a linear correlation with spheroid viability (Figure 6) and dsDNA content (Supplementary Figure S4). Area and volume showed the highest coefficients with 0.7219 and 0.6138, respectively. In contrast, sphericity and convexity showed low R^2^ values (0.1792 and 0.1445) as spheroids did not significantly change even with increased drug and light doses. Convexity showed the lowest coefficient, with only an R^2^ value of 0.0911, with all spheroids showing very similar values regardless of size reduction. Diameter-based parameters showed lower fit values compared to area or volume, with LMinorDTC and Eq. Diameter showing better results compared to LMajorDTC (0.5829 and 0.5536 versus 0.495). DNA content also displayed a similar linear correlation to LDH release, with area showing the highest correlation coefficient. Morphometric parameters showed an improved linear correlation as spheroids were averaged within each condition, demonstrating a relation between viability or DNA content and morphometric parameters area and volume (Supplementary Figure S5). PpIX and PpIX-CD independently showed a similar effect on morphology with equivalent conditions. In both sample types, viability, and total dsDNA% decreased alongside spheroid size, except for sphericity, convexity, and solidity, which were highly inconsistent regardless of treatment conditions (Supplementary Figures 6–9).

## Discussion

### Spheroid Pre-selection for PDT Evaluation

Pre-selection of spheroids has been previously suggested as a method for observing and reducing heterogeneity in sample groups. Zanoni et al. (2016) showed A549 spheroids maintained sphericity (>0.9) over a 25-day period and indicated that this parameter could be used alongside volume to screen samples (Zanoni et al., 2016). High sphericity is a desirable parameter for pre-screening spheroids and is exceptionally important for evaluating PDT as diffusion kinetics of nutrients, oxygen, and drugs are significantly changed by both shape and cell compaction, with irregular/elongated shapes being generally undesirable (Grimes and Currell, 2018). Oxygen consumption drastically changes as cells begin compacting, resulting in an approximately 8-fold increase as spheroid size stabilizes. In turn, this increases the size of the hypoxic zone, a key factor of *in vivo* tumour microenvironments. In addition, Leung et al. (2015) determined sphericity and compactness are highly linked to a uniform solute gradient within MCTS, making them highly important parameters for monitoring (Leung et al., 2015).

Spheroids showed varying degrees of response to PDT combinations. PDT-induced cell death could be observed by the formation of a debris halo surrounding each spheroid. In addition to possible variability in biological assays, the presence of this cellular debris significantly impacted parameter acquisition. Segmentation requires clear images to detect the spheroid contour and improve data extraction. The appearance of apoptotic and dead cell debris as a halo surrounding the spheroid has been previously observed, along with total disruption with elevated drug doses (Mittler et al., 2017). Chen et al. (2014) discussed the variations in manual versus automatic segmentation on spheroid diameters, showing automatic acquisition produces more accurate and consistent measurements (Chen et al., 2014). Spheroid segmentation was also shown to become inaccurate as the spheroid border becomes less defined and the detection of contours is made difficult due to surrounding debris.

### Morphometric Parameters for Estimating Spheroid Damage

PDT-induced spheroid damage was not reflected in many parameters, particularly sphericity, convexity, and solidity. The variation in morphological parameters after drug treatment was also observed by Mittler et al. (2017) (Mittler et al., 2017). Their results closely resembled our observations as sphericity was determined to be independent from spheroid damage after treatment with a variety of chemotherapy drugs. However, image-based analysis is limited by variations in spheroid size and fragility of remaining aggregates. Heavily damaged spheroids were prone to disintegration during debris removal and subsequent well washing. This may have impacted the measurement of more effective treatment combinations such as 3LT PDT using PpIX and PpIX-CD. It is possible that other factors influence treatment outcome in spheroids aside from morphology. Friedrich et al. (2007) demonstrated structurally intact large spheroids contained a large proportion of apoptotic and dead cells. Likewise, the sloughing of outer cell layers with viable cells could be detected through LDH release or DNA quantification (Friedrich et al., 2007). The change in appearance between treated and untreated spheroids can be attributed to a reduction in cell-cell interactions and the loss of adherens junctions (Celli et al., 2014). Size and phenotype dependent response to PDT has been observed in pancreatic ductal adenocarcinoma spheroids embedded within Matrigel and collagen gels, demonstrating the impact of the cell microenvironment on drug responsiveness (Cramer et al., 2017).

Morphological parameters have been shown to be related to drug-responsiveness in spheroids. Thakuri et al. (2019) demonstrated that size-based analysis closely matched traditional assay-based analysis in evaluating various chemotherapy drugs, showing a link between spheroid volume and resazurin reduction (Thakuri et al., 2019). A similar trend was also observed by Ivanov et al. (2014) with the acid phosphatase assay versus volume, though it can only be performed as an endpoint assay due to requiring cell lysis (Ivanov et al., 2014). However, previous estimations calculated spheroid volume assuming spheroids to be perfect spheres instead of the projection method used in AnaSP. Our results showed estimations based on volume tend to have more variability compared to area, which does not rely on assumptions about spheroid morphology. This is in part due to the limitations from working with widefield microscopy images. The use of tools such as ReViSP could be an option for improving the accuracy of volume assessment as they are capable of processing Z-stacks from confocal or light sheet fluorescence microscopes (Piccinini et al., 2015). Nevertheless, it is still unclear if this approach is suitable for HTS spheroid experiments compared to the acquisition of 2D images.

In general, the evaluation of spheroid damage has been done through assays such as LDH release, DNA quantification, adenine triphosphate (ATP) level, or acid phosphatase. Nonetheless, quantitative analysis of spheroid images could also be improved by the addition of fluorescent stains such as live/dead staining to differentiate dead, damaged, and proliferating cells. This could be particularly useful for analyzing variations in similar treatment conditions, as was observed by Sirenko et al. (2015). Additionally, it can also be used to differentiate between compounds and conditions which are cytostatic and cytotoxic.Click or tap here to enter text. However, some assays are difficult to implement as they require careful optimization with spheroid type, size, and experimental conditions. Furthermore, the increase of sample size has been shown to significantly improve statistical confidence during HTS and improve the calculation of effective concentration values for various compounds (Monjaret et al., 2016).

### Limitations of Cancer Spheroids and Light Microscopy

The use of more complex 3D cell models such as cancer organoids and high-content imaging with fluorescent probes has proven to be significantly useful for evaluating PDT parameters. Recent work with organoids has shown drug response is heterogeneous and is not limited to morphological changes, which tend to underestimate PDT effectiveness. 3-D ovarian cancer nodules showed significant variations in treatment effectiveness after PDT under the same conditions, finding a discrepancy between viability and live volume (number of live cells within each nodule) (Anbil et al., 2013). There is evidence that individual nodules react differently to various drugs regardless of morphology. A study with pancreatic 3D cultures demonstrated variations between treatments with various drugs. Etoposide-treated samples showed a non-uniform response, with some maintaining high levels of viability after treatment. In comparison, paclitaxel-treated samples showed a more homogeneous response. Interestingly, morphological disruption also varied depending on the type of drug, with verteporfirin and paclitaxel showing greater changes in comparison to carboplatin, etoposide, and gemcitabine (Celli et al., 2014). Heterogeneity in 3D cell culture models also presents an opportunity for more complex and robust analysis of treatment outcomes. Tumour stroma and non-spheroidal morphologies have been investigated using co-cultures with cancer-associated fibroblasts, demonstrating analysis with spherical shapes is less accurate as they do not closely replicate *in vivo* tumour architecture (Bulin et al., 2017).

The main advantages of light microscopy (LM) are its availability and ease of use. However, it is limited by low image resolution. In contrast, other imaging techniques can produce higher quality images and are more suitable for three-dimensional samples. Light sheet fluorescence microscopy (LSFM) has been shown to be a reliable tool for imaging cancer spheroids. Schmitz et al. (2017) demonstrated LSFM could be used to produce three-dimensional high-quality spheroid models for analysing growth kinetics and inner morphological features (Schmitz et al., 2017). Similarly, Barbier et al. (2016), analysed light-attenuated image stacks obtained by confocal laser scanning microscopy (CSLM) (Barbier et al., 2016). Recently, high-throughput confocal imaging has become available and has been used for morphological analysis of 3D cell cultures. Boutin et al. (2018) demonstrated high-content imaging with U87 spheroids could be achieved within lower timescales (1 h per 384 well plate) at the expense of reduced image quality by increasing slice interval (Boutin et al., 2018).

Although these tools produce highly detailed images, they are unsuitable for larger-scale experiments requiring hundreds of spheroids analysed at specific time points as image processing is severely limited due to hardware constraints. Image analysis using CSLM and LSFM is complex due to the amount of data that is generated; each stack can contain anywhere from 250–400 individual images depending on the slice interval. Volume estimation can greatly vary depending on the type of staining that is performed, sample quality (fixing and staining), and signal intensity. This requires an optimization for each spheroid type and staining protocol, further increasing complexity, as shown by Smyrek and Steltzer (2017) (Smyrek and Stelzer, 2017). Therefore, more precise microscopy tools are ideal for observing small changes in morphology and measure specific morphological parameters more accurately for individual samples.

## Conclusion

This work presented an approach for using spheroid morphometric parameters for screening drug response to various treatment combinations. Spheroid variability was reduced by monitoring morphology during the initial 4-day growth period and pre-selecting samples based on sphericity, diameter, and area. Parameter acquisition was shown to be impacted by image quality and the detection of spheroid contours, with each well requiring rinsing to obtain automatically segmented binary masks. Linear regression analysis showed varying degrees of correlation between parameters area and volume to LDH release and total dsDNA content. In contrast, sphericity and convexity did not show significant change even after extensive spheroid damage. Spheroid analysis using AnaSP could be further improved by using tools to accurately assess spheroid volume (ReViSP) and include fluorescent stains within the workflow. In conclusion, the screening of PDT parameters using spheroid morphology could benefit the evaluation of novel compounds and significantly reduce both time and costs before further *in vitro* or *in vivo* evaluation.

## Data Availability

The raw data supporting the conclusion of this article will be made available by the authors, without undue reservation.

## References

[B1] Aguilar Cosme J. R. Bryant H. E. Claeyssens F. (2019). Carbon Dot-Protoporphyrin IX Conjugates for Improved Drug Delivery and Bioimaging. PLoS ONE 14, e0220210. 10.1371/journal.pone.0220210 31344086 PMC6657888

[B2] Aguilar Cosme J. R. Gagui D. C. Green N. H. Bryant H. E. Claeyssens F. (2021). acsbiomaterials. Washington, DC: ACS Biomaterials Science & Engineering, 1c00690. 10.1021/ACSBIOMATERIALS.1C00690 *In Vitro* Low-Fluence Photodynamic Therapy Parameter Screening Using 3D Tumor Spheroids Shows that Fractionated Light Treatments Enhance Phototoxicity ACS Biomater. Sci. Eng. 34615346

[B3] Aguilar Cosme J. R. (2020). Vitro Photodynamic Therapy Screening with Carbon Dot-Protoporphyrin IX Conjugates. University of Sheffield. Available at: http://etheses.whiterose.ac.uk/26777/(Accessed August 26, 2021).

[B4] Anbil S. Rizvi I. Celli J. P. Alagic N. Pogue B. W. Hasan T. (2013). Impact of Treatment Response Metrics on Photodynamic Therapy Planning and Outcomes in a Three-Dimensional Model of Ovarian Cancer. J. Biomed. Opt. 18, 098004. 10.1117/1.jbo.18.9.098004 24802230 PMC3783041

[B5] Barbier M. Jaensch S. Cornelissen F. Vidic S. Gjerde K. de Hoogt R. (2016). Ellipsoid Segmentation Model for Analyzing Light-Attenuated 3D Confocal Image Stacks of Fluorescent Multi-Cellular Spheroids. PLoS ONE 11, e0156942. 10.1371/journal.pone.0156942 27303813 PMC4909318

[B6] Boutin M. E. Voss T. C. Titus S. A. Cruz-Gutierrez K. Michael S. Ferrer M. (2018). A High-Throughput Imaging and Nuclear Segmentation Analysis Protocol for Cleared 3D Culture Models. Sci. Rep. 8. 10.1038/s41598-018-29169-0 PMC6057966 30042482

[B7] Brüningk S. C. Rivens I. Box C. Oelfke U. ter Haar G. (20202020). 3D Tumour Spheroids for the Prediction of the Effects of Radiation and Hyperthermia Treatments. Sci. Rep. 10 (1 10), 1–13. 10.1038/s41598-020-58569-4 PMC6997397 32015396

[B8] Bulin A.-L. Broekgaarden M. Hasan T. (20172017). Comprehensive High-Throughput Image Analysis for Therapeutic Efficacy of Architecturally Complex Heterotypic Organoids. Sci. Rep. 7 (1 7), 1–12. 10.1038/s41598-017-16622-9 PMC5709388 29192263

[B9] Celli J. P. Rizvi I. Blanden A. R. Massodi I. Glidden M. D. Pogue B. W. (2014). An Imaging-Based Platform for High-Content, Quantitative Evaluation of Therapeutic Response in 3D Tumour Models. Sci. Rep. 4. 10.1038/srep03751 PMC3894557 24435043

[B10] Chen W. Wong C. Vosburgh E. Levine A. J. Foran D. J. Xu E. Y. (2014). High-throughput Image Analysis of Tumor Spheroids: A User-Friendly Software Application to Measure the Size of Spheroids Automatically and Accurately. JoVE. 10.3791/51639 PMC4212916 25046278

[B11] Cho S. Kang D.-K. Sim S. Geier F. Kim J.-Y. Niu X. (2013). Droplet-Based Microfluidic Platform for High-Throughput, Multi-Parameter Screening of Photosensitizer Activity. Anal. Chem. 85, 8866–8872. 10.1021/AC4022067 23937555

[B12] Costa E. C. Moreira A. F. de Melo-Diogo D. Gaspar V. M. Carvalho M. P. Correia I. J. (2016). 3D Tumor Spheroids: an Overview on the Tools and Techniques Used for Their Analysis. Biotechnol. Adv. 34, 1427–1441. 10.1016/j.biotechadv.2016.11.002 27845258

[B13] Cramer G. M. Jones D. P. El-Hamidi H. Celli J. P. (2017). ECM Composition and Rheology Regulate Growth, Motility, and Response to Photodynamic Therapy in 3D Models of Pancreatic Ductal Adenocarcinoma. Mol. Cancer Res. 15, 15–25. 10.1158/1541-7786.MCR-16-0260 27671335 PMC5381935

[B14] Curto V. F. Ferro M. P. Mariani F. Scavetta E. Owens R. M. (2018). A Planar Impedance Sensor for 3D Spheroids. Lab. Chip 18, 933–943. 10.1039/c8lc00067k 29459934

[B15] Das V. Fürst T. Gurská S. Džubák P. Hajdúch M. (2016). Reproducibility of Uniform Spheroid Formation in 384-Well Plates. J. Biomol. Screen. 21, 923–930. 10.1177/1087057116651867 27226477

[B16] Friedrich J. Eder W. Castaneda J. Doss M. Huber E. Ebner R. (2007). A Reliable Tool to Determine Cell Viability in Complex 3-D Culture: The Acid Phosphatase Assay. J. Biomol. Screen. 12, 925–937. 10.1177/1087057107306839 17942785

[B17] Friedrich J. Seidel C. Ebner R. Kunz-Schughart L. A. (2009). Spheroid-based Drug Screen: Considerations and Practical Approach. Nat. Protoc. 4, 309–324. 10.1038/nprot.2008.226 19214182

[B18] Grimes D. R. Currell F. J. (2018). Oxygen Diffusion in Ellipsoidal Tumour Spheroids. J. R. Soc. Interf. 15, 20180256. 10.1098/rsif.2018.0256 PMC6127169 30111663

[B19] Hari N. Patel P. Ross J. Hicks K. Vanholsbeeck F. (2019). Optical Coherence Tomography Complements Confocal Microscopy for Investigation of Multicellular Tumour Spheroids. Sci. Rep. 9, 1–11. 10.1038/s41598-019-47000-2 31332221 PMC6646385

[B20] Imamura Y. Mukohara T. Shimono Y. Funakoshi Y. Chayahara N. Toyoda M. (2015). Comparison of 2D- and 3D-Culture Models as Drug-Testing Platforms in Breast Cancer. Oncol. Rep. 33, 1837–1843. 10.3892/or.2015.3767 25634491

[B21] Ivanov D. P. Parker T. L. Walker D. A. Alexander C. Ashford M. B. Gellert P. R. (2014). Multiplexing Spheroid Volume, Resazurin and Acid Phosphatase Viability Assays for High-Throughput Screening of Tumour Spheroids and Stem Cell Neurospheres. PLoS ONE 9, e103817. 10.1371/journal.pone.0103817 25119185 PMC4131917

[B22] Ivascu A. Kubbies M. (2007). Diversity of Cell-Mediated Adhesions in Breast Cancer Spheroids. Int. J. Oncol. 31, 1403–1413. 10.3892/ijo.31.6.1403 17982667

[B23] Kepp O. Galluzzi L. Lipinski M. Yuan J. Kroemer G. (2011). Cell Death Assays for Drug Discovery. Nat. Rev. Drug Discov. 10, 221–237. 10.1038/nrd3373 21358741

[B24] Kessel S. Cribbes S. Bonasu S. Rice W. Qiu J. Chan L. L.-Y. (2017). Real-time Viability and Apoptosis Kinetic Detection Method of 3D Multicellular Tumor Spheroids Using the Celigo Image Cytometer. Cytometry 91, 883–892. 10.1002/cyto.a.23143 28618188

[B25] Kim T.-H. Mount C. W. Gombotz W. R. Pun S. H. (2010). The Delivery of Doxorubicin to 3-D Multicellular Spheroids and Tumors in a Murine Xenograft Model Using Tumor-Penetrating Triblock Polymeric Micelles. Biomaterials 31, 7386–7397. 10.1016/j.biomaterials.2010.06.004 20598741

[B26] Lazzari G. Vinciguerra D. Balasso A. Nicolas V. Goudin N. Garfa-Traore M. (2019). Light Sheet Fluorescence Microscopy versus Confocal Microscopy: in Quest of a Suitable Tool to Assess Drug and Nanomedicine Penetration into Multicellular Tumor Spheroids. Eur. J. Pharmaceutics Biopharmaceutics 142, 195–203. 10.1016/j.ejpb.2019.06.019 31228557

[B27] Leung B. M. Lesher-Perez S. C. Matsuoka T. Moraes C. Takayama S. (2015). Media Additives to Promote Spheroid Circularity and Compactness in Hanging Drop Platform. Biomater. Sci. 3, 336–344. 10.1039/c4bm00319e 26218124

[B28] Lou X. Kim G. Koo Y. Kopelman R. Yoon E. (2010), High-throughput of Photodynamic Therapy (PDT) Screening from Multiple Parameter Assays of 1,000 Different Conditions in a Single Chip.

[B29] Mehta G. Hsiao A. Y. Ingram M. Luker G. D. Takayama S. (2012). Opportunities and Challenges for Use of Tumor Spheroids as Models to Test Drug Delivery and Efficacy. J. Controlled Release 164, 192–204. 10.1016/j.jconrel.2012.04.045 PMC3436947 22613880

[B30] Mittler F. Obeïd P. Rulina A. V. Haguet V. Gidrol X. Balakirev M. Y. (2017). High-content Monitoring of Drug Effects in a 3D Spheroid Model. Front. Oncol. 7. 10.3389/fonc.2017.00293 PMC5732143 29322028

[B31] Monjaret F. Fernandes M. Duchemin-Pelletier E. Argento A. Degot S. Young J. (2016). Fully Automated One-step Production of Functional 3D Tumor Spheroids for High-Content Screening. J. Lab. Autom. 21, 268–280. 10.1177/2211068215607058 26385905

[B32] Perche F. Torchilin V. P. (2012). Cancer Cell Spheroids as a Model to Evaluate Chemotherapy Protocols. Cancer Biol. Ther. 13, 1205–1213. 10.4161/cbt.21353 22892843 PMC3469478

[B33] Piccinini F. (2015). AnaSP: A Software Suite for Automatic Image Analysis of Multicellular Spheroids. Comput. Methods Programs Biomed. 119, 43–52. 10.1016/j.cmpb.2015.02.006 25737369

[B34] Piccinini F. Tesei A. Arienti C. Bevilacqua A. (2015). Cancer Multicellular Spheroids: Volume Assessment from a Single 2D Projection. Comput. Methods Programs Biomed. 118, 95–106. 10.1016/j.cmpb.2014.12.003 25561413

[B35] Rühland S. Wechselberger A. Spitzweg C. Huss R. Nelson P. J. Harz H. (2015). Quantification of *In Vitro* Mesenchymal Stem Cell Invasion into Tumor Spheroids Using Selective Plane Illumination Microscopy. J. Biomed. Opt. 20, 1. 10.1117/1.jbo.20.4.040501 25839427

[B36] Sant S. Johnston P. A. (2017). The Production of 3D Tumor Spheroids for Cancer Drug Discovery. Drug Discov. Today Tech. 23, 27–36. 10.1016/j.ddtec.2017.03.002 PMC5497458 28647083

[B37] Schmitz A. Fischer S. C. Mattheyer C. Pampaloni F. Stelzer E. H. K. (2017). Multiscale Image Analysis Reveals Structural Heterogeneity of the Cell Microenvironment in Homotypic Spheroids. Sci. Rep. 7. 10.1038/srep43693 PMC5334646 28255161

[B38] Sirenko O. Mitlo T. Hesley J. Luke S. Owens W. Cromwell E. F. (2015). High-Content Assays for Characterizing the Viability and Morphology of 3D Cancer Spheroid Cultures. ASSAY Drug Dev. Tech. 13, 402–414. 10.1089/adt.2015.655 PMC4556086 26317884

[B39] Smyrek I. Stelzer E. H. K. (2017). Quantitative Three-Dimensional Evaluation of Immunofluorescence Staining for Large Whole Mount Spheroids with Light Sheet Microscopy. Biomed. Opt. Express 8, 484. 10.1364/boe.8.000484 28270962 PMC5330556

[B40] Thakuri P. S. Gupta M. Plaster M. Tavana H. (2019). Quantitative Size-Based Analysis of Tumor Spheroids and Responses to Therapeutics. Assay Drug Dev. Tech. 17, 140–149. 10.1089/adt.2018.895 PMC6599382 30958703

[B41] Vinci M. Gowan S. Boxall F. Patterson L. Zimmermann M. Court W. (2012). Advances in Establishment and Analysis of Three-Dimensional Tumor Spheroid-Based Functional Assays for Target Validation and Drug Evaluation. BMC Biol. 10. 10.1186/1741-7007-10-29 PMC3349530 22439642

[B42] Zanoni M. Piccinini F. Arienti C. Zamagni A. Santi S. Polico R. (2016). 3D Tumor Spheroid Models for *In Vitro* Therapeutic Screening: A Systematic Approach to Enhance the Biological Relevance of Data Obtained. Sci. Rep. 6. 10.1038/srep19103 PMC4707510 26752500

