# Supplementary material for: Morphological Response in Cancer Spheroids for Screening Photodynamic Therapy Parameters
Source: Front Mol Biosci. 2021 Nov 18;8:784962. doi: 10.3389/fmolb.2021.784962 (PMC8637197; doi:10.3389/fmolb.2021.784962)
Supplement: Supplementary file 1 [file DataSheet1.PDF]

# *Supplementary Material*

**Supplementary Table 1**. List of morphological parameters extracted from MCTS using AnaSP.

| Parameter | Explanation |
|---|---|
| Area (*A*) | Total number of pixels in foreground |
| Volume (*V*) | Volume estimated from segmented image projection (ReViSP) |
| Perimeter (*P*) | Total number of pixels in outer border |
| LMajorDTC | Maximum length of axis through centre of spheroid mass |
| LMinorDTC | Minimum length of axis through centre of spheroid mass |
| Eq. Diameter | Diameter corresponding to a circle with equivalent area |
| Convexity | Degree of spheroid curvature. |
| Solidity | Degree of spheroid compaction after growth. (Area/Convexity*Area) |
| Sphericity | Degree of similitude to a perfect sphere. |

**Supplementary Table 2**. Automatic segmentation shows variations from manual segmentation in AnaSP.

| **Segmentation** | **Manual (n=8)** | | **Automatic (n=8)** | | **% Variation** |
|---|---|---|---|---|---|
| **Parameter** | **Mean** | **CV%** | **Mean** | **CV%** | |
| Area | 44193 | 12.19 | 61594 | 2.83 | **+28.25** |
| Convexity | 0.981 | 0.82 | 0.861 | 3.60 | **-13.92** |
| Eq. Diameter | 236.752 | 6.22 | 278.958 | 0.94 | **+15.13** |
| LMajorDTC | 253.736 | 7.14 | 297.455 | 1.98 | **+14.70** |
| LMinorDTC | 217.917 | 5.56 | 259.162 | 0.75 | **+15.91** |
| Perimeter | 815.333 | 5.64 | 1180.667 | 7.65 | **+30.95** |
| Solidity | 0.979 | 0.10 | 0.937 | 1.28 | **-4.51** |
| Sphericity | 0.912 | 0.66 | 0.775 | 4.13 | **-17.69** |
| Volume | 6694064 | 16.44 | 11132698 | 3.51 | **+39.87** |

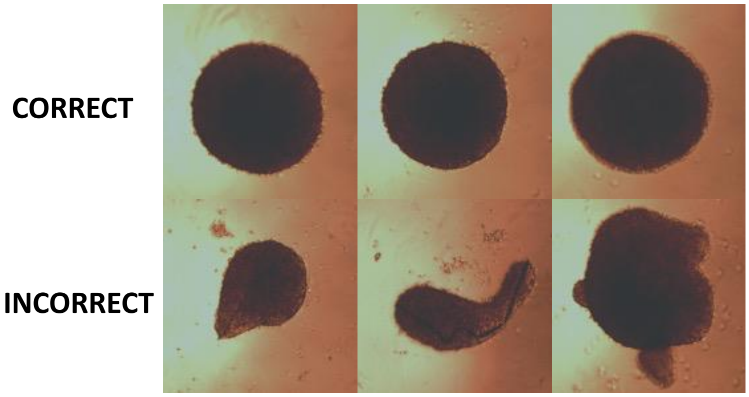


**Supplementary Fig. 1 – Spheroid morphology should become quasispherical after several days of growth.**

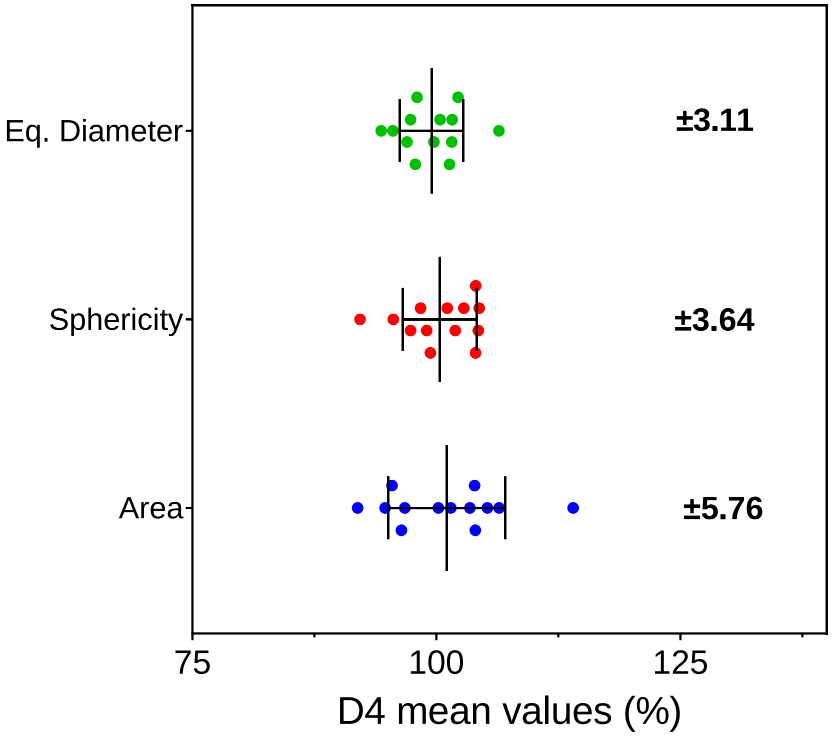


**Supplementary Fig. 2 – Area, sphericity, and equivalent diameter were used to reduce spheroid viability prior to *in vitro* experiments.** The standard deviation was reduced by removing outlying samples: area (8.41 to 5.76), sphericity (4.75 to 3.64), and equivalent diameter (8.03 to 3.11).

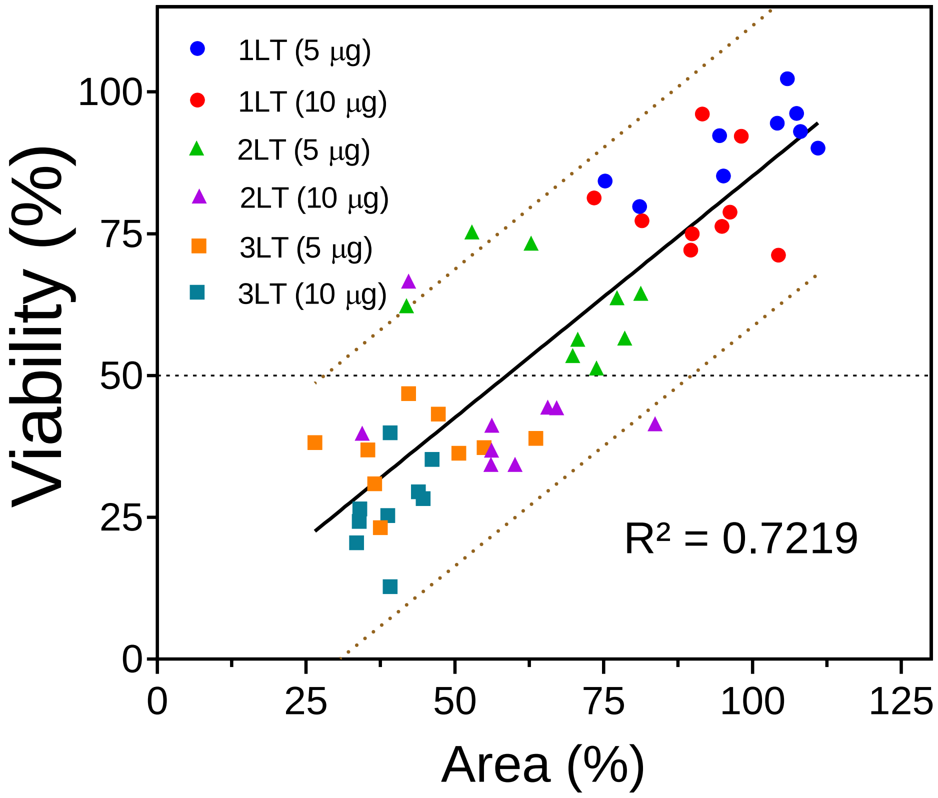


**Supplementary Fig. 3 - Light fractionated treatments showed variation with individual samples within groups.** PpIX 1LT groups (top right) and 3LT groups (bottom left) appear to be more tightly clustered compared to 2LT.

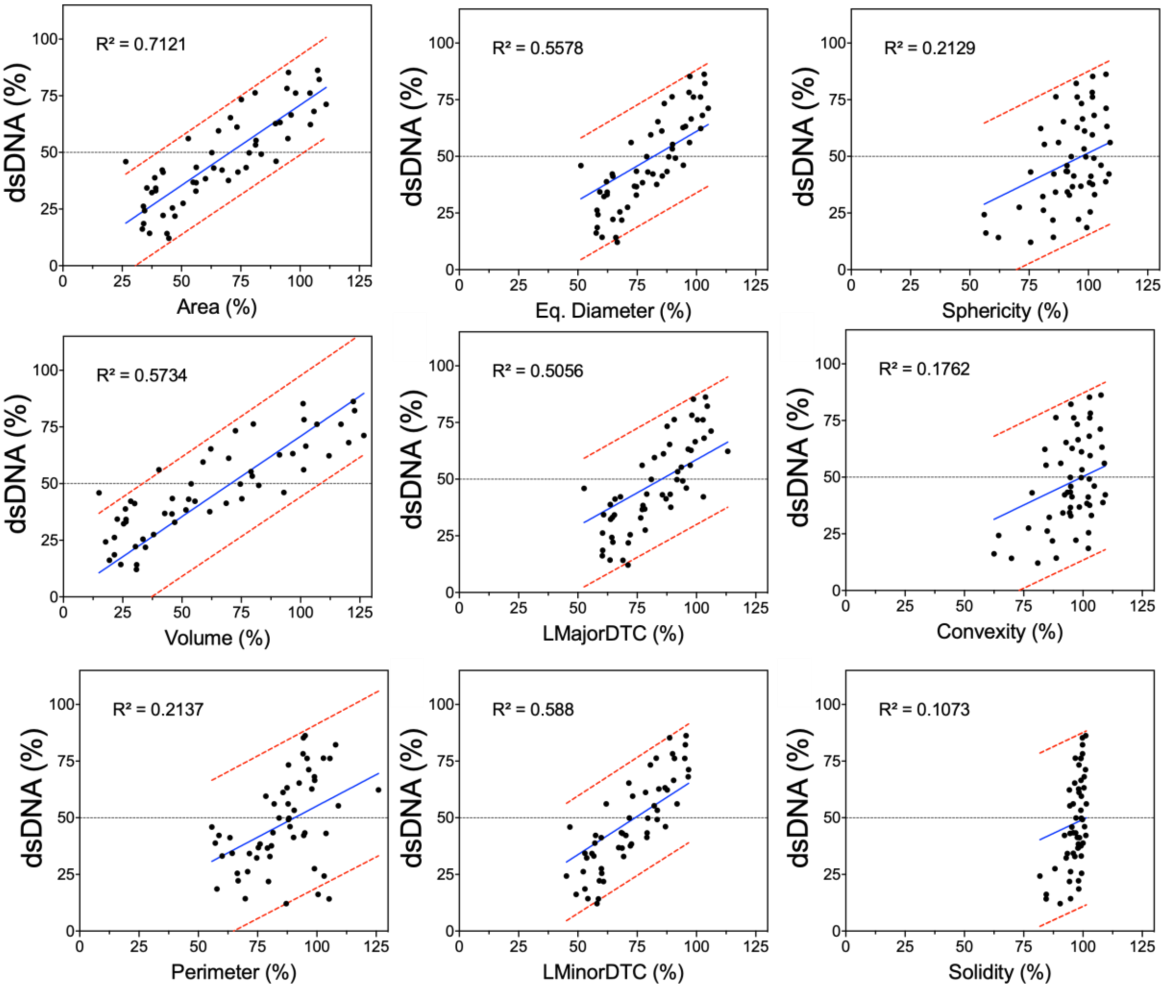


**Supplementary Fig. 4 – Spheroid morphology is linked to various morphometric parameters.** Acquired parameters show varying degrees of linear correlation with total dsDNA based on control spheroids at the same time point (24 hours after light activation). Each dot represents a single spheroid after PDT with PpIX or PpIX-CD. The regression line is shown in blue, and the 95% prediction intervals are shown in red. Widefield images of spheroids after different combinations of PDT treatments are shown on the right (PpIX, 10 µg/ml). (N=3, n=6)

| | | | Area | Convexity | Eq. Diam | LMajorDTC | LMinorDTC | Perimeter | Solidity | Sphericity | Volume |
|---|---|---|---|---|---|---|---|---|---|---|---|
| PpIX | Viability | Single | 0.7219 | 0.1445 | 0.5536 | 0.495 | 0.5829 | 0.2507 | 0.0911 | 0.1792 | 0.6138 |
| | | Grouped | 0.9467 | 0.1934 | 0.6591 | 0.6136 | 0.6953 | 0.4431 | 0.1021 | 0.2534 | 0.9414 |
| | dsDNA | Single | 0.7121 | 0.1762 | 0.5578 | 0.5056 | 0.588 | 0.2137 | 0.1073 | 0.2129 | 0.5734 |
| | | Grouped | 0.9599 | 0.2151 | 0.7175 | 0.6664 | 0.7582 | 0.4959 | 0.1157 | 0.2816 | 0.8356 |
| PpIX-CD | Viability | Single | 0.77 | 0.0534 | 0.7133 | 0.6462 | 0.7591 | 0.4275 | 0.0752 | 0.0692 | 0.5445 |
| | | Grouped | 0.9133 | 0.1018 | 0.7752 | 0.7177 | 0.8292 | 0.59 | 0.0852 | 0.1481 | 0.7331 |
| | dsDNA | Single | 0.7333 | 0.00004 | 0.684 | 0.6459 | 0.719 | 0.4643 | 0.0449 | -0.003 | 0.4661 |
| | | Grouped | 0.90004 | 0.0987 | 0.7459 | 0.6876 | 0.7955 | 0.555 | 0.0833 | 0.1471 | 0.7316 |

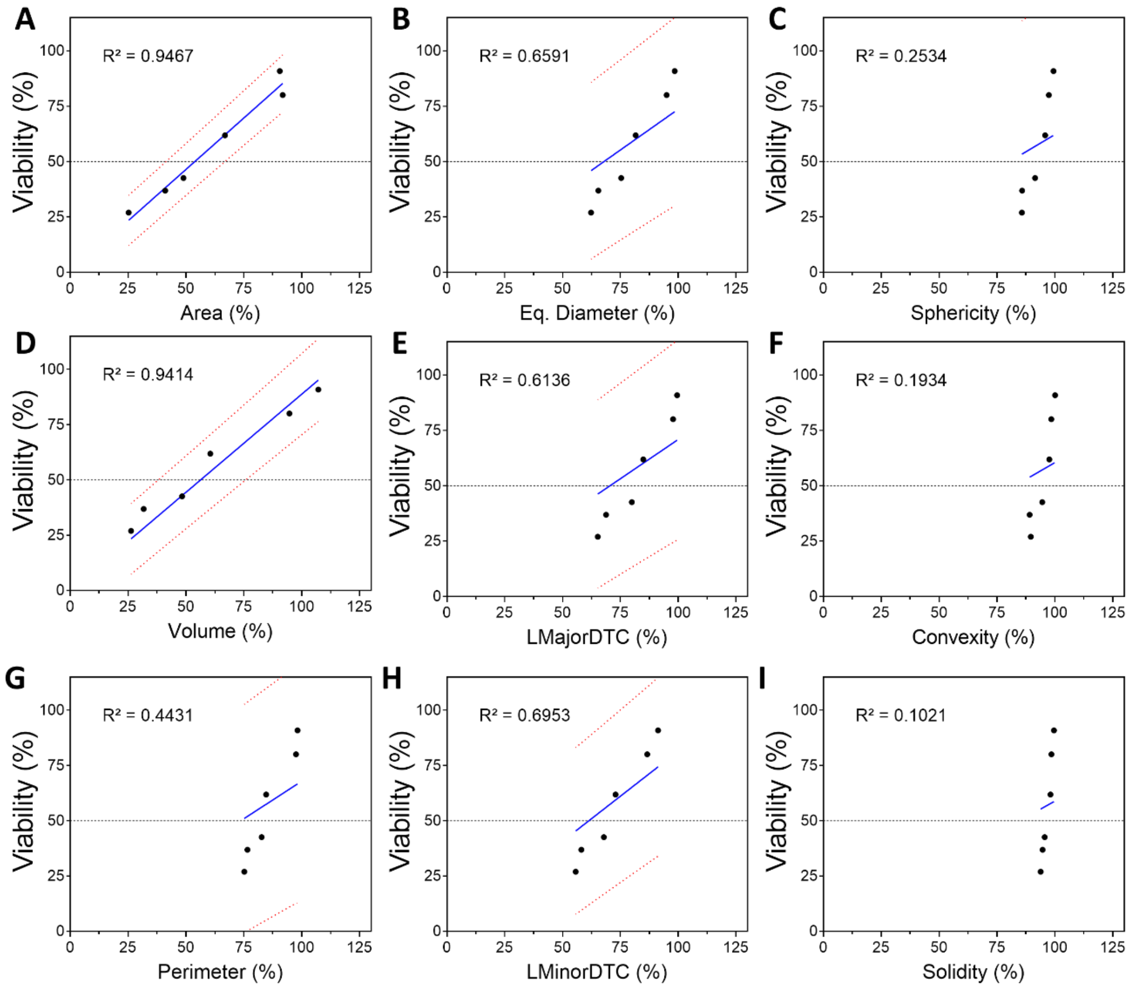


**Supplementary Fig. 5 - $R^2$ values for morphometric parameters improve with treatment averages**. Single spheroids showed lower correlation values compared to the averages of treatment conditions. Graphs show the in correlation between viability and morphometric parameters with PpIX in contrast with Fig. 6. Area, volume, equivalent diameter and LMinorDTC showed the greatest change from previous results.

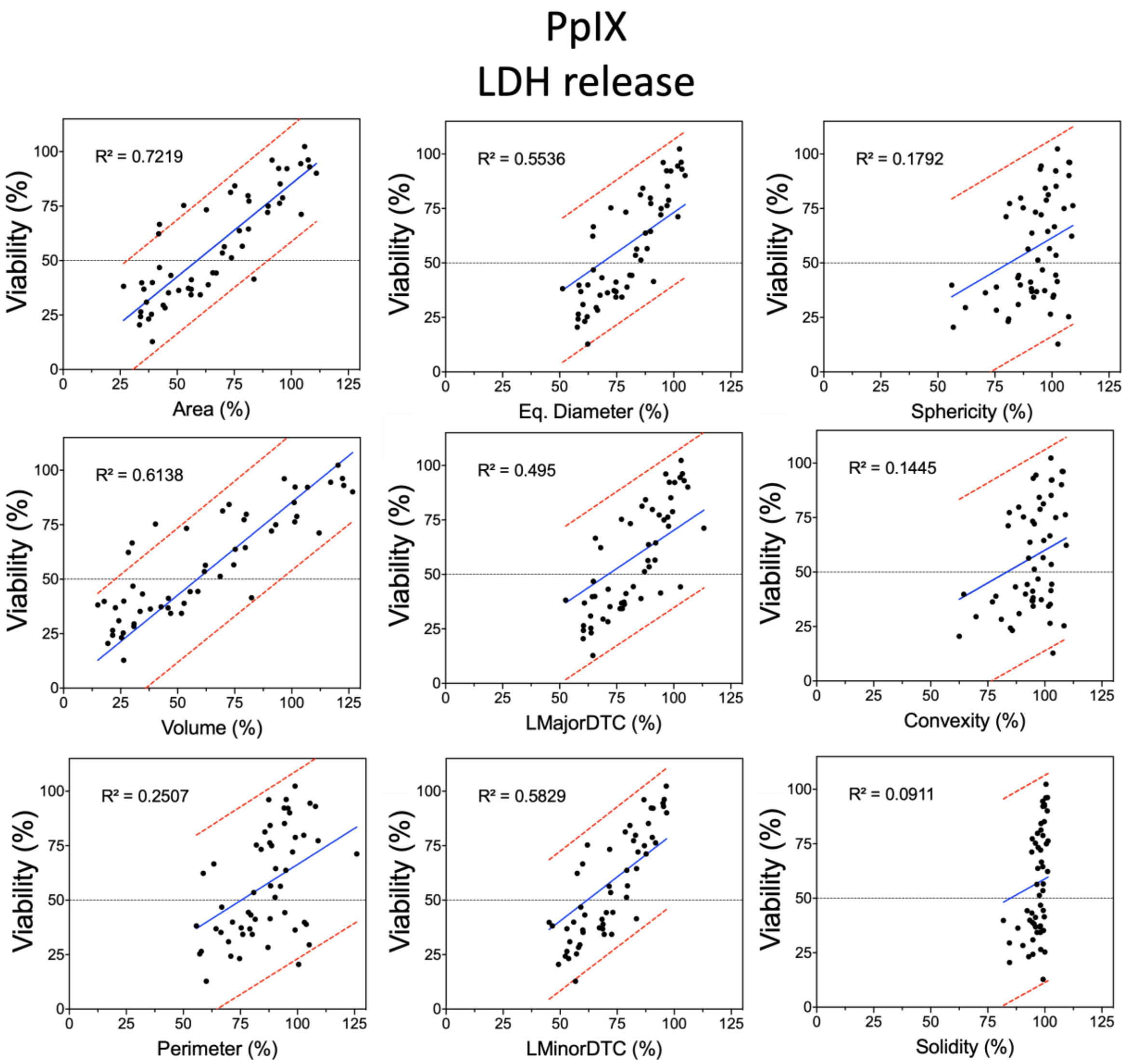


**Supplementary Fig. 6 – Comparison of LDH release and morphometric parameters.**

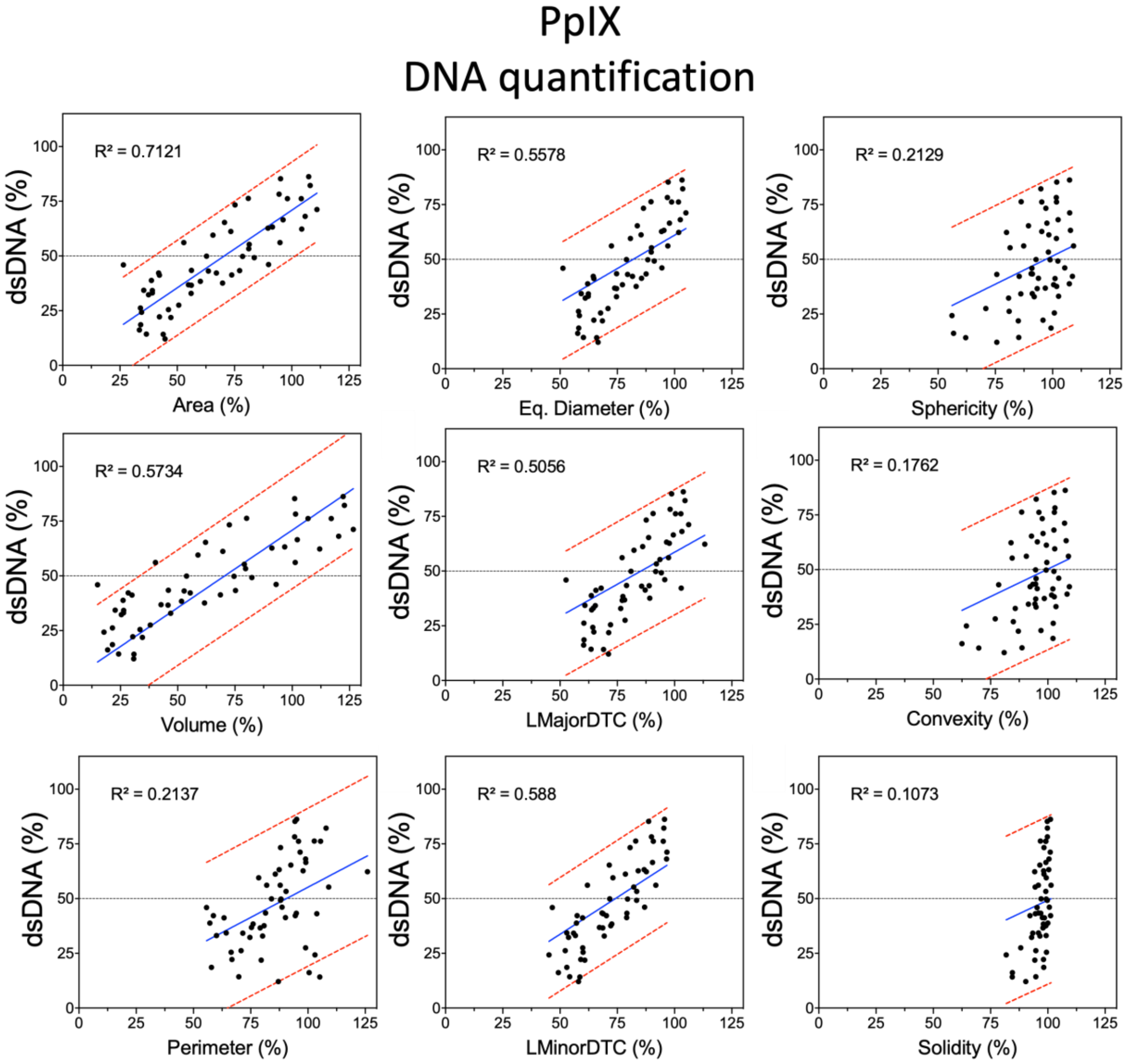


**Supplementary Fig. 7 – Comparison of dsDNA content and morphometric parameters.**

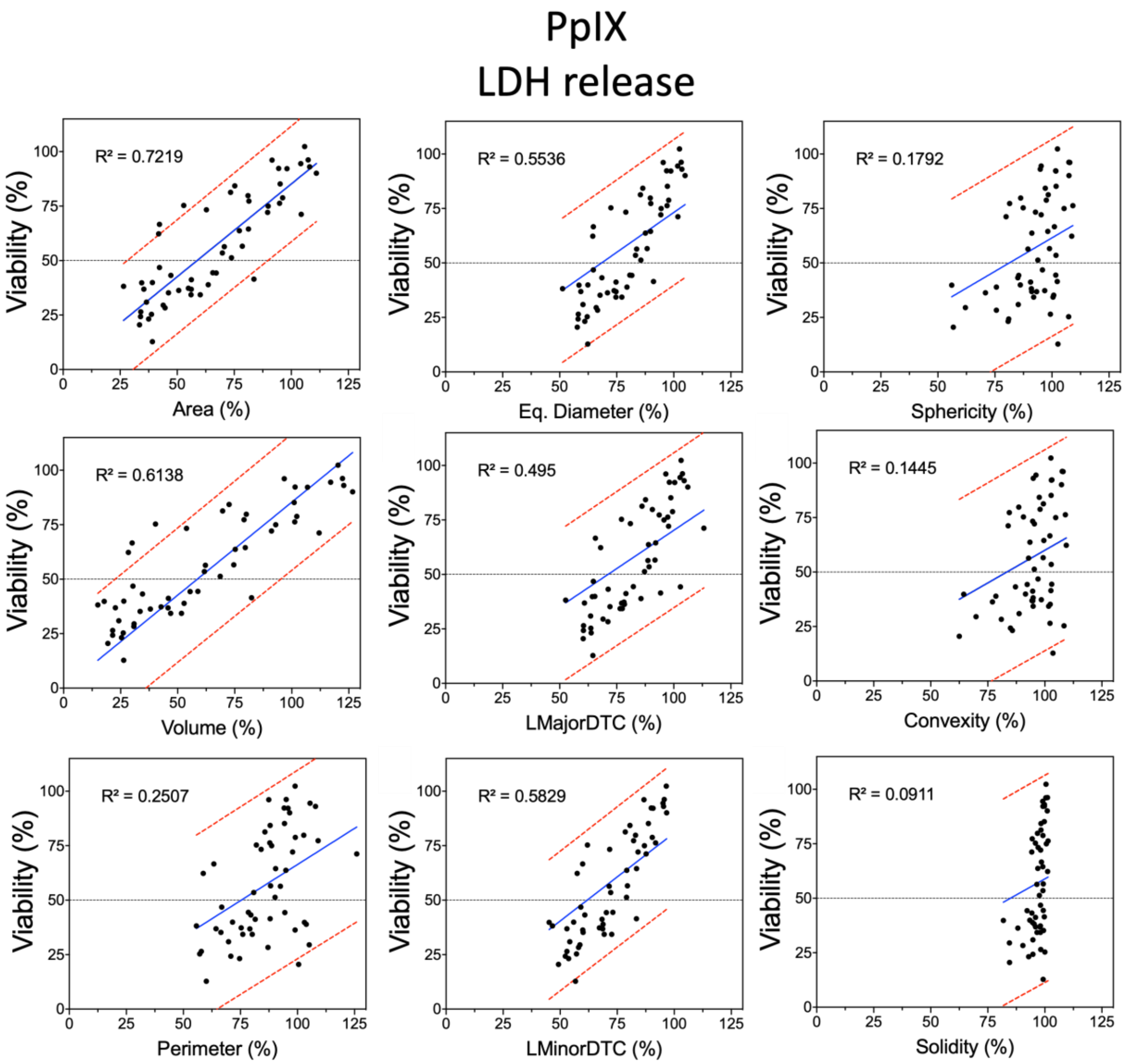


**Supplementary Fig. 8 – Comparison of LDH release and morphometric parameters.**

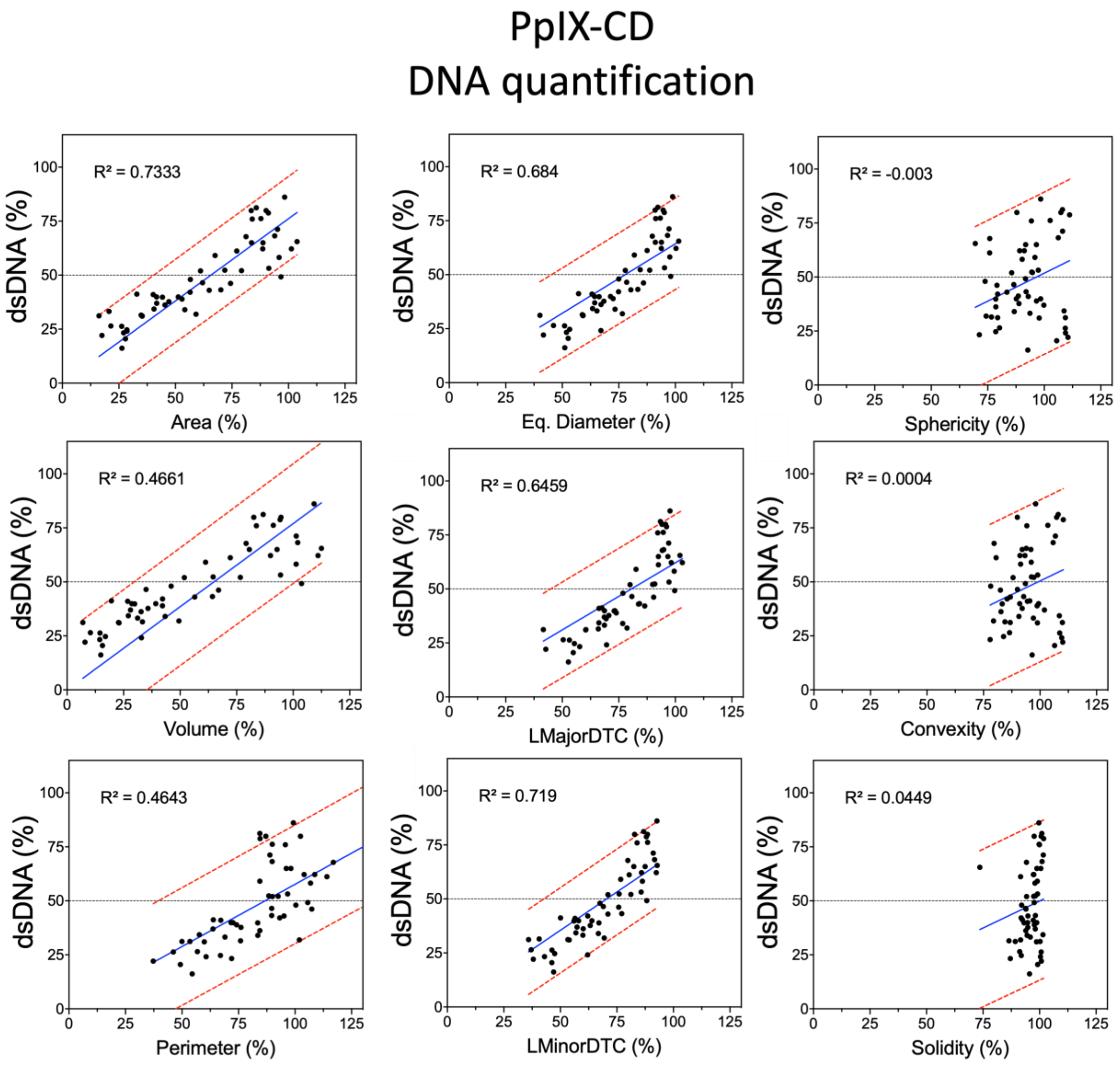


**Supplementary Fig. 9 – Comparison of dsDNA content and morphometric parameters.**
